# Phylogenetic Analysis of Torque Teno Virus in Hepatitis C Virus Infected Patients in Shiraz

**DOI:** 10.5812/hepatmon.6133

**Published:** 2012-07-30

**Authors:** Azra Kenar Koohi, Mehrdad Ravanshad, Manouchehr Rasouli, Shahab Falahi, Ashraf Baghban

**Affiliations:** 1 Department of Virology, Faculty of Medical Sciences, Tarbiat Modares University, Tehran, IR Iran; 2 Alborzi Clinical Microbiology Research Center, Nemazee Hospital, Shiraz, IR Iran; 3 Department of Microbiology, School of Medicine, Ilam University of Medical Sciences, Ilam, IR Iran

**Keywords:** Hepatitis C, Prevalence, Shiraz

## Abstract

**Background:**

Torque teno virus (TTV) was the first human Circoviridae detected in a Japanese patient with unknown hepatitis in 1997. Subsequently, several studies performed to evaluate different aspects of Torque teno virus pathogenesis.

**Objectives:**

The present study aimed to determine dominant genotype of Torque teno virus in chronic hepatitis disease using 5΄-UTR sequence among patients infected by hepatitis C virus in Shiraz – Iran.

**Patients and Methods:**

The study conducted in 240 patients with chronic hepatitis C from Prof. Alborzi Clinical Microbiology Research Center. The presence of Torque teno virus DNA and its genotype in plasma was assessed by nested polymerase chain reaction using two primer sets for 5΄-UTR and N22 regions. Phylogenetic analysis was performed based on 5΄-UTR region.

**Results:**

DNA of Torque teno virus was detected in 220 out of 240 (92 %) patients with chronic hepatitis C by the use of 5΄-UTR primer based PCR method and in 12 out of 240 (5%) by the use of N22 primer. Based on phylogenetic analysis it was shown that the Dominant genotype in this study was 11. Genotypes 1, 3, 17, and 22 were also detected. Some sequences could not be classified to a specific genotype.

**Conclusions:**

The prevalence of Torque teno virus DNA in patients with chronic hepatitis C disease by the use of 5΄-UTR primer appeared to be higher compared to that revealed by N22 primer. We observed five genotypes among hepatitis C chronic patients in our study.

## 1. Background

In 1997 torque teno virus (TTV) was first described in Japanese patients with non-A-G transfusion-transmitted hepatitis of unknown etiology [1][2][3]. Visualization of TTV derived from clinical samples of infected individuals showed a un-enveloped virus with single- stranded circular DNA genome of negative polarity, 3.4-3.9 Kb in length, and several open reading frames [2][3]. TTV is currently classified in Circoviridae family. Despite other DNA viruses, TTV exhibits a drastic wide sequence diversity [2]. Further studies showed that accumulated TTV DNA titers correlate with the level of aminotransferase in the patients [4]. Several clinical findings together with detected TTV DNA in 47 % of patients with fulminant hepatitis and in 46 % of patients with chronic liver disease of unknown etiology support the proposal that TTV may deserve partly as a possible agent for acute and chronic liver diseases of unknown etiology [3]. TTV is distributed in more than 50 % of normal human population throughout the world. Although it was first detected in patients infected by transfusion , recent studies suggested other ways of transmission [2]. The current TTV phylogeny reveals extensive sequence heterogeneity to the extent that a general agreement on the typing of different isolates may not be concluded from the literature. In phylogenetic studies, more than 30 genotypes were identified, [5] some of them with different geographic distribution [6]. Phylogenetic assignments are defined on the basis of N22 region and distance criterion of > 0.30 [7]. Major genotyping studies used sequences of N22 region and reported that genotypes 1, 2, and 3 are highly prevalent worldwide [5].The UTR region is comparatively well conserved and contains several segments with an identity greater than 90 % indicating that high variation is not tolerated in these regions [8]. UTR-5΄ primer can detect most of TTV genotypes while for N22 primer this detection is limited [9][10]. Earlier studies proposed that due to the complexity and unlimited results of N22 region, it seems that phylogenetic analysis based on UTR sequence is more accurate and reliable [11]. Also, one study reported that phylogenetic classification based on the N22 region is unreliable [7]. Several researches showed the presence of this virus in patients infected by HCV and believed that TTV titer is correlated with severity of carcinoma attributed to HCV and therefore it can be used as a prognostic predictor for the outcome of chronic HCV infection [12][13]. In recent years, several studies performed to determine the prevalence, modes of transmission, and clinical relevance of TT virus [14]. However, no data are available in regards to circulating TTV genotypes in Iran.

## 2. Objevtives

The present study constitutes the first genomic characterization of TTV in Iran and reports 30 isolates of the virus from Iranian patients infected by chronic HCV whom sequenced and analyzed to determine their genotypes.

## 3. Patients and Methods

### 3.1. Patient, Sample and Isolation of Viral Nucleic Acid

Population of this study consisted of individuals referred to Professor Alborzi Clinical Microbiology Research Center, Namazi Hospital, Shiraz, Iran due to HCV infection. During 2006-2009, 240 HCV infected blood samples were collected and centrifuged immediately, to separate sera in aliquot parts and cryopreserve at -70 °C until analysis. All HCV infected patients of study population were tested by PCR on 5΄-UTR and N22 regions for TTV infection. This study was approved by the committee of human research publication and ethics of Tarbiat Modares University and consent was obtained from the patients. Briefly, DNA was isolated from 140 µL serum of collected peripheral blood mononuclear cells (PBMC) of patients by the use of an in-house protocol and a QIAamp Viral DNA Mini Kit (QIAGEN) with some modification in manufacturer’s instruction. Nucleic acids were eluted from the filter column using 50 µL of nuclease-free elution buffer and preserved at -70°C until analysis.

### 3.2. Primer Design

Primers selected for this study were shown in Table 1 and Table 2. These primers bind to 5΄-UTR and N22 regions of TT virus. In regards to the locations of designed primers, TTV amplicons were formed having lengths of 220 and 270 nucleotides for 5΄-UTR and N22 regions, respectively. Furthermore, only one existing sequence with NC_002076. Two accession numbers were used as references. Primers for PCR were designed by the use of Mega 4 NCBI (National Center for Biotechnology Information) and were based on alignments of different TTV genome sequences from GenBank. Based on examination software, primer sets used in this study amplified approximately all known TTV variants. Gene Runner, NCBI Blast, Oligo analyzer3, and Oligo6 software were used to design and compare different sets of primers. In order to obtain the most appropriate combination, results of virtual amplifications were evaluated and different pairs of primers were selected (Table 1, 2).

**Table 1:** Primer Sequences for 5’-UTR Region of torque teno virus

**Primer Name**	**Detected Region**	**Primer Sequence**
NG 054	3-22 (sense, outer and inner)	5′- TTT GCT ACG TCA CTA ACC AC -3′
NG1471	211-233 (Antisense, outer)	5′ -GCC AGT CCC GAG CCC GAA TTG CC -3′
NG1321	204-223 (antisense, inner)	5′-AGC CCG AAT TGC CCC TTG AC -3′

**Table 2:** Primer Sequences for N22 Region of torque teno virus

**Primer Name**	**Detected region**	**Primer Sequence**
NG059	1900-1923 (sense, outer and inner)	5′-ACA GAC AGA GGA GAA GGC AAC ATG-3′
NG061	1915-1936 (Antisense, outer)	5′-GGC AAC ATG YTR TGG ATA GAC TGG-3′
NG063	2161-2185 (antisense, inner)	5′-CTG GCA TTT TAC CAT TTC CAA AGT T-3′

### 3.3. Semi Nested PCR Detection for TTV DNA

In the first step, seven confirmed positive control samples were obtained and applied to set up the procedure. (Samples courtesy of Dr. Zohre Sharifi, Tehran Blood Bank Organization). The first and second rounds of TTV nested PCR reactions for 5΄-UTR region contained 5 μL of template DNA, 0.5μL or 0.3 μL (10pM stock) of each amplification primer, respectively, 0.5 μL of dNTP (10 mM stock), 2.5 μL of Taq DNA polymerase (Cinnagen,Tehran, Iran), 0.5mM of MgCl2, and 2.5 μL of 10× buffer (500mM of KCl and Tris-HCl, pH=8.4). PCR amplifications were performed as follows: initial denaturation at 95 °C, 5 min and 35 cycle’s of 40 sec each at 94 °C, 45 sec at 60 °C, and 50 sec at 72 °C, with a final 7 min extension at 72 °C. The PCR products were separated by electrophoresis in 2 % agarose gels and visualized after staining by ethidium bromide. The final cycling program was employed as follows: initial denaturation at 95 °C, 5 min and 35 cycles of 50 sec each at 94 °C, 40 sec at 65 °C, and 40 sec at 72 °C, with a final 3 min extension at 72 °C. Program used for the second PCR round was designed similar to the first round PCR except for the number of cycles that were reduced to 25 and reaction template was selected equal to 2 μL from the first round product. The amplification products of the second PCR round were 220 bp. Semi nested N22 PCR: In the first round, outer primers NG059 and NG061 and in the second round NG059 and NG063 were used. TTV DNA Amplification conditions based on N22 primer were initial denaturation at 95 °C, 7 min and , 35 cycles, of 94°C at 30sec, 57°C at 45sec, 72°C at 50sec with a final 7 min extension at 72°C. Program used for the second PCR round was similar to the first round PCR except for the number of cycles reduced to 20 and reaction template was selected equal to 2 μL from first round product. The amplification products of the second PCR round were 271 bp. More technical data were published elsewhere [15].

### 3.4. Sequences From the Database

In total, 30 sequences of TTV Genotypes were obtained from GenBank and applied to compare sequences of the isolates in the study. The accession numbers of sequences were reported as following: AF247137.1, ABO54647.1, AF234023.1, ABO28668.1, AF261761.1, AF515694.1, AF515685.1, AF122916.1, AF122921.1, AB025946.1, AB017613.1, ABO54648.1, AF122919.1, AF122914.3, AF247138.1, AB017610.1, NC002076.1, GU179314.1, GU179313.1, GU179302.1, GU179307.1, GU179303.1, GU179311.1, GU179309.1, GQ466173.1, GQ466174.1, GU179340.1, GU179335.1, GU179341.1, GU179338.1, GU179325.1, GU179339.1, GU179317.1, GU179322.1.

### 3.5. Phylogenetic Analysis

Phylogenetic analysis was performed, based on TTV 5’-UTR nucleotide sequence and TTV reference sequences. All of the results were edited by Bioedit (Ibis Biosciences, USA), and ClustalX (EMBL-EBI, UK) software for multiple alignments. Genetic distance was evaluated using Kimura-two-parameter matrix [16]. Phylogenetic trees were constructed by neighbor-joining (NJ) method [17]. Bootstrap resampling was carried out on 100 replicates to ensure the reliability of the tree. In order to further proper analysis of sequences, the maximum-parsimony method was also used [18].

## 4. Results

**Figure 1:**
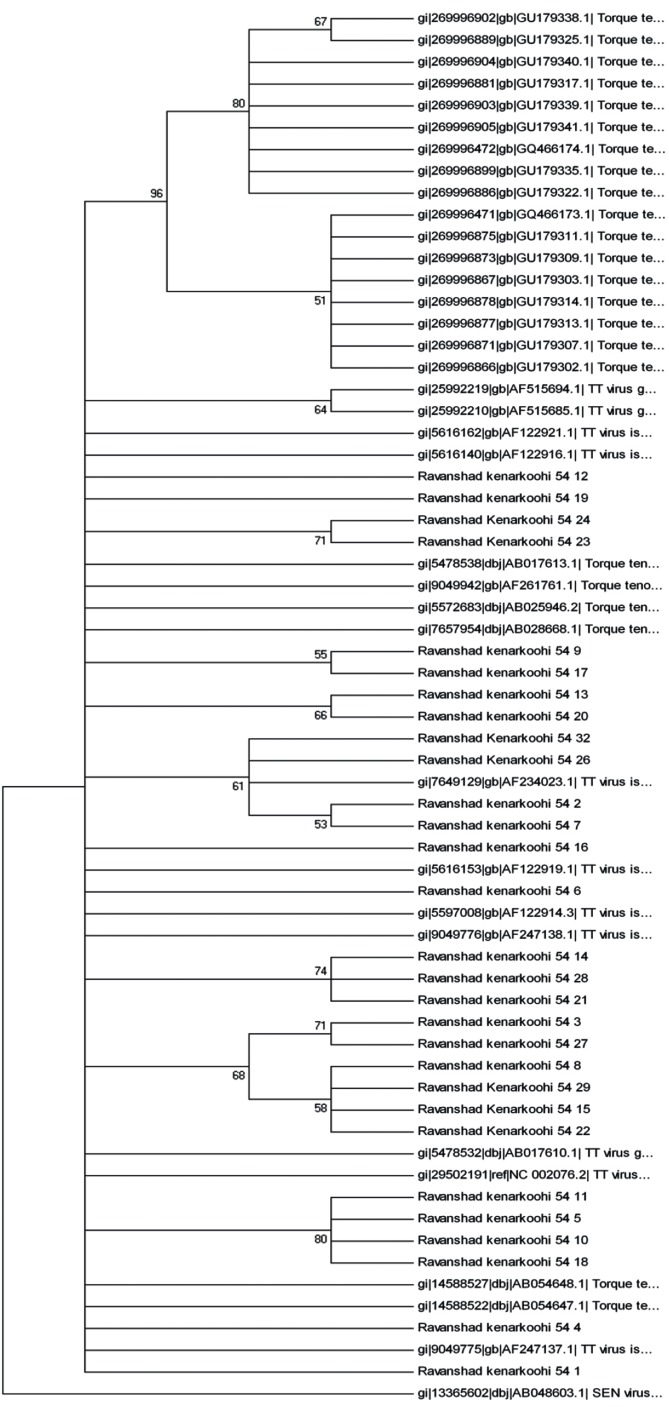
Constructed Phylogenetic Tree by the Use of Kimura Two-Parameter Matrix and Neighbor-Joining Method, Based on 5’-UTR Sequence (220nt- residues3- 233). References: 5’-UTR sequence from TTV genomes were identified by their GenBank accession numbers

**Figure 2:**
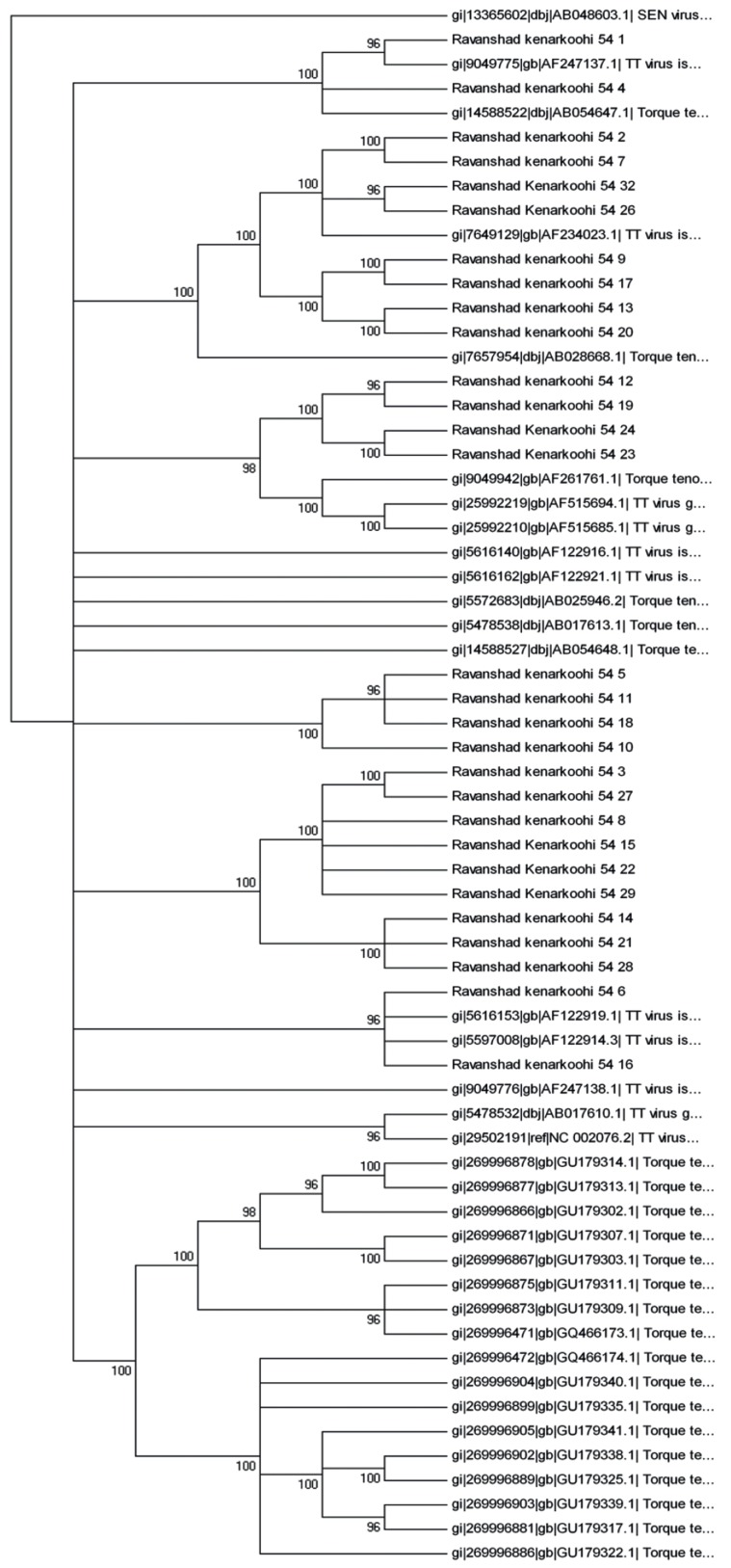
Constructed Phylogenetic Tree by the Use of Maximum Parsimony Method, Based on 5’-UTR Sequence (220nt-residues3- 233) References: 5’-UTR Sequence from TTV genomes were identified by their GenBank Accession Numbers

TTV-DNA was detected in 220 out of 240 (92 %) patients with chronic hepatitis C disease by 5΄-UTR primer based PCR method and in 12 out of 240 (5 %) of patients by N22 primer. All positive samples [5] detected by N22 primer were also detected as positive by 5΄-UTR primer. Phylogenetic analysis was performed and 5 genotypes (1, 3, 11, 17,and 22) were identified (Figure 1 and Figure 2). Results obtained in two trees were relatively similar. Some sequences did not match to the genotypes mentioned in the gene bank.

## 5. Discussion

TTV was originally isolated from a patient with cryptogenic hepatitis and assumed that TTV may associated with hepatitis, although to date a causative effect has not been established [14]. in order to clear pathology of TTV infection, further studies should be performed to evaluate prevalence, genotyping, and pathogenicity of TTV infection. TTV has been found to be extremely common in humans with high worldwide prevalence. Currently, PCR is the only available assay for detection of TTV virus [18]. However, efficient and complete detection of all genotypes depends on the selected region and primer sensitivity. Recently used primers are derived from either N22 or 5’-UTR regions for DNA amplification. The primers of N22 region could only detect few TTV genotypes but 5’-UTR primers allowed amplification of almost all known genotypes of TTV [11]. The present results demonstrated that TTV infection is frequently detected by 5’-UTR primers in chronic HCV patients. Ninety two percent prevalence detected by 5’-UTR primers study group of chronic HCV patients but it was lower when N22 primers were employed (5 %). Based on the current investigation it was concluded that primers for N22 which is widely used for detection the virus as well as 5’-UTR which we have also used, would report prevalence ranging from 5 to 92 % due to their sensitivity. High prevalence of TTV observed by using 5’-UTR primers might be due to frequent preservation of 5’-UTR region among different genotypes. Several studies have reported that the prevalence of TTV may vary within a single population according to the gene region ampliﬁed and the primers used. In Brazil, TTV prevalence among Brazilian blood donors using different primers demonstrated different values ranging from 11.9 %to 50.5 % [5].The same result was observed in Saudi Arabia studies from 19 % to 100 % by the use of 5’-UTR and N22 primers, respectively [8]. Because of a very high genetic diversity of virus, the reported prevalence in different studies and populations has been continuously repeated and was affected by sensitivity of selected primers as well as the selected viral region [10]. Early studies on TTV phylogenetic analysis were investigated on N22 region because it was assumed that the region would exhibit enough diversity for phylogenetic analysis but TTV DNA revealed an extensive genetic diversity in spite of other DNA viruses, when detected by PCR. Also, TTV classification was problematic within this divergent region that was resulted in controversial and unreliable observations. Our study also showed that it may be difficult to classify TTV isolates based on extensive sequence heterogeneity in this region and therefore concluded phylogenetic analysis by the use of 5’-UTR, a more preserved region. To date, there was little or no information reported about the prevalence and genotype distribution of human TT virus in Iranian patients infected by HCV. Thirty strains of TTV DNA were isolated from Iranian patients with chronic HCV disease and a stretch of 220 nt from 5’-UTR region were successfully sequenced. In order to achieve more accurate results, we employed two methods (neighbor-joining and maximum- parsimony) and compared the analysis of phylogenetic tree. Some of TTV strains had been classified because others did not correspond to gene bank genotypes. They may constitute of new branches in phylogenetic tree. To achieve exact classification it is better to investigate full length genome. we classified the genotypes as 1, 3,11,17, and 22. Sequence analysis of 30 of 5’-UTR positive isolates revealed that Genotype 11 was significantly more prevalent among Iranian patients; however, it was reported that worldwide dominant genotypes of TTV were 1, and 3 [5]. In addition to genotype 11, genotypes 1, 3, 17, 22 were also identified. Results observed by maximum parsimony tree were in consistent with genotype sequences available in database. It seems that classification of TTV by maximum parsimony method may be more precise than that by neighbor-joining method. We observed five genotypes among chronic HCV patients in Iran. In order to identify more genotypes, we probably should investigate more samples. Another reason attributed to such result might be selection of patients from different regions of the country. This study indicates that TTV is widespread among chronic HCV patients in Iran. Further studies require determining the correlation between HCV and TTV.
